# The prevalence of outgrowing non‐priority legume allergies in children

**DOI:** 10.1111/pai.70269

**Published:** 2025-12-22

**Authors:** Ali Atwah, Kate Swan, Adam T. Fox, Ru‐Xin Foong

**Affiliations:** ^1^ Department of Pediatrics, Faculty of Medicine King Abdulaziz University Rabigh Saudi Arabia; ^2^ Department of Women and Children's Health (Pediatric Allergy), School of Life Course Sciences, Faculty of Life Sciences and Medicine King's College London London UK; ^3^ Children's Allergy Service, Evelina London Children's Hospital Guy's and St Thomas' Hospital London UK

**Keywords:** bean allergy, chickpea allergy, food allergy resolution, IgE‐mediated food allergy, legume cross‐reactivity, lentil allergy, non‐priority legumes, pea allergy

## Abstract

**Background:**

Non‐priority legume (NPL) allergies are increasing, creating significant concerns due to the widespread use of these foods. This study aims to evaluate the natural history of NPL allergy in children and identify predictive factors associated with their resolution.

**Methods:**

A retrospective review of medical records of pediatric patients (Age 5–15 Years) from the Pediatric Allergy Department at Evelina London Children's Hospital (from July 2023 to June 2024) who had an IgE‐mediated allergy to any NPLs (lentils, chickpeas, peas, and beans). Children with a suggestive history of NPL allergy with a positive skin prick test (SPT) ≥8 Mm, specific immunoglobulin E (sIgE) ≥15 kUA/L and/or positive oral food challenge were included.

**Results:**

Two hundred and three children were included; the median age was 10 years (62.6% male), and median duration of follow‐up was 7 years. 6.3% of all children with food allergies were allergic to NPLs, with lentils being most common. Kaplan–Meier analysis showed varying resolution rates by age: 2.2% for lentils, 4.9% for chickpeas, 8.3% for peas, and 13% for beans by age 5. By age 10, these rates increased to 11.3%, 8.8%, 20.1%, and 24.5%, respectively, and by age 15, they increased to 21% for lentils, 19.3% for chickpeas, 23.5% for peas, and 32.9% for beans. Children who had low SPT and sIgE results were more likely to outgrow their legume allergy.

**Conclusion:**

NPL allergy represents a significant disease burden and the rate of resolution by age 15 years was 20%–32.9%, similar to legumes such as peanut.

**Abbreviations**
EAR: European Anaphylaxis Registry
EOE: eosinophilic oesophagitis
FA: food allergy
FSA: Food Standards Agency
NPL: non‐priority legume
ns‐LTPs: nonspecific lipid transfer proteins
OAS: oral allergy syndrome
OFC: oral food challenge
SIgE: specific immunoglobulin E
SPT: skin prick test
UK: United Kingdom

**Key message**

Early childhood allergy to non‐priority legumes (NPL), such as lentils, peas, chickpeas, and beans, is not uncommon. This study highlights their resolution patterns, with many children outgrowing their NPL allergy, ranging between 19.3% and 32.9%. Those with lower SPT/sIgE levels are more likely to outgrow their allergy.

## 1 BACKGROUND

Food allergies (FA) are a major public health concern, particularly among children, and they can significantly impact quality of life. In recent decades, the prevalence of FA has increased globally, reaching 4%–5% in England, and even higher prevalence rates in other countries.^1^ Accurate FA diagnosis and identifying children who may outgrow their allergies are crucial for effective management and ensuring that the fewest number of foods are avoided.

In the United Kingdom (UK), the Food Standards Agency (FSA) mandates the labelling of 14 specified food allergens.^2^, ^3^ Legumes are categorized into priority and non‐priority groups based on their inclusion as a major food allergen that requires labelling. The priority group includes peanuts, lupine, and soybeans, which have been extensively investigated in the scientific literature.^2^, ^3^, ^4^ The non‐priority legume (NPL) group includes beans, peas, lentils, chickpeas, fava beans, fenugreek, tamarindus indica, and cowpea. The FSA labelling regulations do not require a declaration for non‐priority legumes in food products.^3^

The prevalence of NPL allergies is higher in Mediterranean countries and Asian countries such as India where they are considered among the most common foods responsible for FA^5^, ^6^ These legumes can trigger IgE‐mediated allergic reactions ranging from mild skin reactions to life‐threatening anaphylaxis. Moreover, many studies identified cases of anaphylaxis to NPLs, specifically lentils and peas, often in conjunction with an allergy to peanuts or other legumes.^7^, ^8^, ^9^ Although uncommon, food hypersensitivity via inhalation can occur in sensitized individuals and may lead to allergic symptoms. Inhaled food allergens may trigger a range of symptoms, including respiratory manifestations (such as rhinitis and asthma), cutaneous reactions (like urticaria), and, in rare cases, systemic anaphylaxis^10^, ^11^

Notably, the prevalence of NPL allergies appears to be increasing worldwide, necessitating more attention.^4^ The reasons for this are complex and may be related to epigenetic factors influenced by lifestyle, pollution, climate change, and other environmental factors.^4^ In addition, legume consumption has increased because it is a good source of protein, the popularity of vegetarianism and plant‐based diets, and the increased use of pre‐prepared foods. These factors may promote increased sensitisation among the population.^4^, ^12^ NPL allergies have not been researched as extensively as other legumes, despite their increasing prevalence. A study from the European Anaphylaxis Registry (EAR) identified NPL as elicitors of interest in food‐induced anaphylaxis.^13^ These highlight the need for a better understanding of NPL natural history and likelihood of resolution is required.

Various studies of other foods, including legumes such as soy and peanut, have shown that FA is not always a lifelong problem^14^ The aim of this study was to assess the prevalence of NPL allergy resolution in children and the potential factors influencing the likelihood of outgrowing these allergies in a UK population of children.

## 2 METHODOLOGY

### 2.1 Study design

This was a retrospective review‐based study that used existing medical records from children seen in a tertiary pediatric allergy department at Evelina London Children's Hospital, UK to collect data on NPL allergy. This study was registered with the Guy's and St Thomas' Hospital audit department (16673).

### 2.2 Study population

The study focused on children aged five and older diagnosed with NPL allergies including chickpeas, lentils, peas, and beans only. The study population included any child with a history suggestive of an IgE‐mediated allergy to NPLs with positive skin prick test (SPT) results, specific Immunoglobulin E (sIgE) tests, and/or oral food challenge (OFC). SPT was performed using fresh food of NPLs and sIgE testing was performed using the Thermofisher platform (see Appendix S1 for further SPT and sIgE details). In children with a history of relevant NPL allergen exposure, an SPT ≥8 mm and/or a sIgE ≥15 kUA/L was considered confirmatory of allergy. Children with suspected allergy, but no clear clinical history and allergy tests that were below these cutoff values were offered an OFC.

The study excluded any child who was eligible for an OFC but did not undergo the procedure, either due to parental preference or the child's lack of interest in consuming NPLs. In addition, children with incomplete records or insufficient follow‐up data, non‐IgE‐mediated allergy to legumes, or with G6PD deficiency were excluded.

### 2.3 Non‐priority legumes

This study defines NPLs specifically as lentils, chickpeas, peas, and beans, which were the primary focus of our analysis. Although the broader category of non‐priority legumes may include fenugreek, cowpea, and fava bean, these were not part of the current study.

### 2.4 Data collection

Medical records were reviewed and data was collected on patient demographics, allergy history, test results for legumes (SPT and sIgE), and information on allergic disorders such as atopic dermatitis, asthma, allergic rhinitis, eosinophilic esophagitis, and oral allergy syndrome. Allergy resolution status was determined by OFC or consumption of the legume without allergic reactions at ages 5, 10, and 15 years. The study's primary outcome was the prevalence of legume allergy resolution, which was defined as a negative OFC or tolerance to NPL without allergic reactions. Persistent allergy was defined as patients who had SPT or sIgE levels above the cutoffs, patients who continued to experience allergic reactions to the NPL despite SPT or sIgE levels below the cutoffs, or positive OFC.

A secondary outcome was the identification of any predictors of allergy resolution, including allergy tests (SPT and sIgE) at baseline. An additional outcome was the nature and severity of any reported reactions, including those which occurred through inhalant exposure.

### 2.5 Statistical analysis

The analysis for this research was conducted using SPSS software (IBM Corp. Released 2012. IBM SPSS Statistics for Windows, Version 21.0. Armonk, NY: IBM Corp). Qualitative variables were expressed using percentages, while median and interquartile ranges (25th–75th percentiles) were used for quantitative variables. A Kaplan–Meier analysis assessed patterns of allergy resolution over time. The chi‐square test was used to determine significant predictors for outgrowing these allergies, analyzing the association between categorical variables and allergy status (either persistent or resolved). The Mann–Whitney test evaluated differences in median SPT and sIgE levels between resolved and persistent allergies, while the Kruskal–Wallis test compared quantitative variables among children with single, dual, and multiple NPL allergies. When significant differences were identified, post hoc pairwise comparisons were conducted. A *p* value <.05 was considered statistically significant. A sensitivity analysis was conducted to address any missing SPT or sIgE data (see Table S1 for further details).

## 3 RESULTS

A total of 3246 records of patients aged 5–15 years with IgE‐mediated FA, who presented to the hospital between July 2023 and June 2024, were reviewed. Of these, 203 (6.3%) children fulfilled the inclusion criteria of IgE‐mediated NPL allergy. Among participants, the prevalence of co‐morbid atopic conditions included 201 (99%) children who had FA to other foods, 192 (94.6%) had atopic dermatitis, 160 (78.8%) had allergic rhinitis, 141 (69.5%) had asthma, 18 (8.9%) had oral allergy syndrome (OAS), and 9 (4.4%) had eosinophilic oesophagitis (EoE). Of those children diagnosed with NPL allergies, 139 children (68.5%) had lentil allergy, 84 (41.4%) had pea allergy, 83 (40.9%) had chickpea allergy, and 46 (22.7%) had bean allergy. The majority with NPLs were male (62.6%). The median age at diagnosis of first NPL allergy was 2 years (IQR: 1–4) and the median duration of follow‐up was 7 years (IQR: 4–9) (Table 1).

**TABLE 1:** Demographic and clinical characteristics of children with a diagnosis of non‐priority legumes.

Variables		Frequency	Percent (%)
		*N* = 203	
Gender	Female	76	37.4
	Male	127	62.6
Age: Median (25th–75th IQR)		10 (7, 13)	
Age at diagnosis: Median (25th–75th IQR)		2 (1, 4)	
Duration of follow‐up: Median (25th–75th IQR)		7 (4, 9)	
Legume allergy	Lentil	139	68.5
	Chickpea	83	40.9
	Pea	84	41.4
	Bean	46	22.7
Diagnosis	Clinical history (+ SPT and/or IgE)	201	99
	Positive oral food challenge	2	1
Type of first reaction	Erythematous rash and Itchiness	63	31
	Multiple symptoms (anaphylaxis symptoms excluded)	42	20.7
	Hives	32	15.8
	Angioedema	26	12.8
	Vomiting	18	8.9
	Anaphylaxis	14	6.9
	Throat Itchiness	6	2.9
	Cough	2	1
Other food allergies	Yes	201	99
History of atopic disorders	Atopic dermatitis	192	94.6
	Allergic rhinitis	160	78.8
	Asthma	141	69.5
	Oral allergy syndrome	18	8.9
	Eosinophilic esophagitis	9	4.4
Number of self‐report of reaction from inhalant exposure		22	10.8
Ever anaphylaxis		42	20.7

Regarding the nature of the initial allergic reactions experienced, erythematous rash/itching (31%), hives (15.8%), angioedema (12.8%), vomiting (8.9%), throat itching (3%), cough (1%), and anaphylaxis (6.9%). 20.7% of the children exhibited more than one symptom (excluding anaphylaxis symptoms). Notably, 20.7% reported a history of anaphylaxis to an NPL at some point, and 10.8% self‐reported a reaction from an inhalant exposure to the NPL (Table 1).

Across single, dual, and multiple NPL allergy groups, male predominance was observed (63.3%, 59.6%, and 64.3%, respectively). The median age at diagnosis was 3 years (IQR 2–4) in single, 2 years (IQR 1–4) in dual, and 2 years (IQR 1–3) in multiple allergy groups, with post hoc analysis confirming significance between the single and multiple groups. Atopic dermatitis was common in all groups (94.5%, 98.1%, and 90.5%, respectively), as was allergic rhinitis (77.1%, 82.7%, and 78.6%, respectively) and asthma (65.5%, 78.4%, and 69%, respectively). Furthermore, other food allergies were reported in 100% of children in the single group, 97.6% of the dual group, and 98.1% of the multiple group although none were statistically significant. However, a history of anaphylaxis was significantly higher in children with multiple NPL allergies (35.7%) compared to those with dual (25%) and single (12.8%) NPL allergies. Median follow‐up duration was 7 years (IQR 4–8), 7.5 years (IQR 5.8–10), and 7 years (IQR 4.5–12), respectively (Table 2).

**TABLE 2:** Comparison of clinical characteristics among children with single, dual, and multiple non‐priority legume allergies.

Factors	Single		Dual		Multiple		χ^2^
	*N*	%	*N*	%	*N*	%	*p* Value
Male	69	63.3	31	59.6	27	64.3	0.27 .8
Age at diagnosis Median (25th–75th IQR)	3 (2, 4)*		2 (1, 4)		2 (1, 3)*		0.005 .003* (Post‐hoc)
Duration of follow‐up Median (25th–75th IQR)	7 (4, 8)		7.5 (5.75, 10)		7 (4.5, 12)		.07
Atopic dermatitis	103	94.5	51	98.1	38	90.5	2.5 .28
Allergic rhinitis	84	77.1	43	82.7	33	78.6	0.67 .72
Asthma	72	65.5	40	78.4	29	69	2.77 .25
Other food allergies	109	100	41	97.6	51	98.1	2.39 .3
Ever anaphylaxis	14	12.8	13	25	15	35.7	10.5 .005

* Post‐hoc pairwise comparisons were performed to identify significant differences between groups.

**FIGURE 1:**
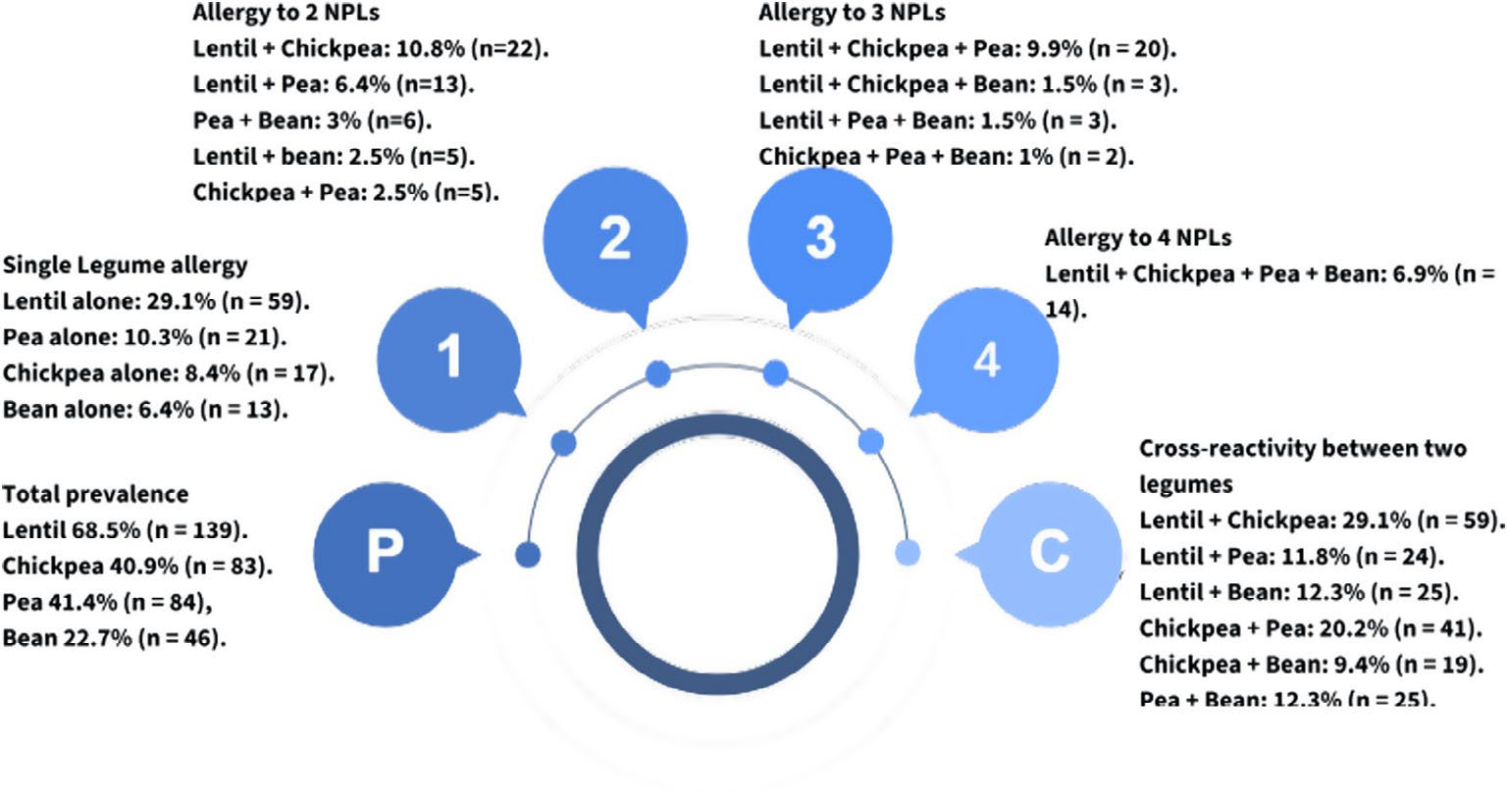
Prevalence and cross‐reaction of non‐priority legume allergies in the study population.

**FIGURE 2:**
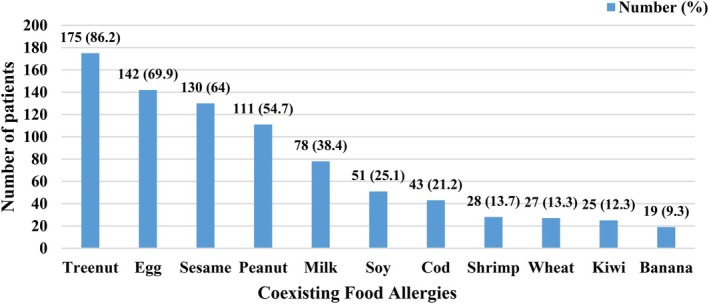
Prevalence of coexisting food allergies among children with NPL allergies.

Single NPL allergy prevalence was 29.1% for lentil, 8.4% for chickpea, 10.3% for pea, and 6.4% for bean. Dual NPL allergies included lentil–chickpea (10.8%), lentil–pea (6.4%), pea–bean (3%), and chickpea–pea or lentil–bean (2.5% each). There were 6.9% of children who were allergic to all four legumes (Figure 1). Nearly all children had other non‐NPL food allergies—tree nuts (86.2%), egg (69.9%), sesame (64%), peanut (54.7%), milk (38.4%), soy (25.1%), fish (21.2%), and shrimp (13.7%) (Figure 2).

**FIGURE 3:**
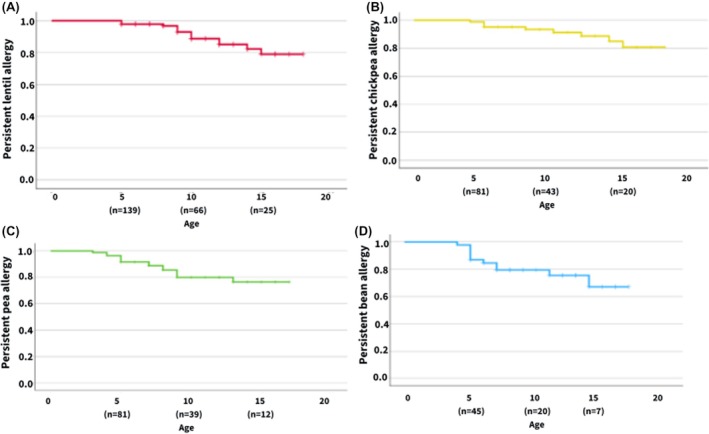
Legume allergy resolution over time. The graph represents the Kaplan–Meier Curve showing the outcome of NPL Allergies by Age with Number of Patients at Each Age Interval. (A) Lentil allergy resolution curve, (B) Chickpea allergy resolution curve, (C) Pea allergy resolution curve, and (D) Bean allergy resolution curve.

We examined the longitudinal resolution of legume allergies among children. At age 5, 2.2% for lentils, 4.9% for chickpeas, 8.3% for peas, and 13% for beans had outgrown their legume allergy. By age 10, the resolution rates were as follows: 11.3% for lentil, 8.8% for chickpea, 20.1% for pea, and 24.5% for bean. At age 15, the resolution rates were 21% for lentil, 19.3% for chickpea, 23.5% for pea, and 32.9% for bean (Figure 3). The total numbers of children who outgrew their legume allergy—either by passing an OFC or by consuming the legume without experiencing a reaction—were as follows: 14 for lentil (6 through consumption without reaction and 8 with a negative OFC), 9 for chickpea (5 through consumption and 4 with a negative OFC), 15 for pea (5 through consumption and 10 with a negative OFC), and 11 for bean (4 through consumption and 7 with a negative OFC) (Table 3).

**TABLE 3:** Outcomes of oral food challenges at any time or tolerance of legume via consumption, with corresponding SPT and IgE results in children with non‐priority legume allergies.

Allergy status	Non‐priority legume		Lentil	Chickpea	Pea	Bean
Persistent	No OFC (SPTor sIgE > study cut‐off)	*n*	89	53	42	14
		SPT Median (25th–75th IQR) IgE kUA/L median (25th‐75th IQR)	SPT:10 (7, 13) IgE: 19 (16.8, 27.5)	SPT: 7 (3, 8) IgE: 20 (15.5, 32)	SPT: 6 (3, 8) IgE: 22 (16, 69)	SPT: 8.5 (8,10) IgE: 21.5 (16, 31)
	No OFC (Clinical reaction)	*n*	34	20	26	21
		SPT Median (25th–75th IQR) IgE kUA/L median (25th‐75th IQR)	SPT: 4 (0, 6) IgE: 5.3 (2.2, 6.6)	SPT: 3.5 (2, 5) IgE: 6.5 (4.3, 7.9)	SPT: 4 (3, 5) IgE: 6.4 (1.1, 8.5)	SPT: 3 (3,4) IgE:2.5 (0.8, 4.1)
	Positive OFC (at any time)	*n*	5	2	4	1
		SPT Median (25th–75th IQR) IgE kUA/L median (25th‐75th IQR)	SPT: 0 (0, 3) IgE: 6.5 (4.6,8.5)	SPT: 2.5 (2,3) IgE: 1.9 (1.8,2)	SPT: 3 (3,5) IgE:2.6 (1.8, 4.2)	IgE (0.26)
Total OFC		*n*	13	6	14	8
Resolved	Negative OFC	*n*	8	4	10	7
		SPT Median (25th–75th IQR) IgE kUA/L median (25th‐75th IQR)	SPT: 2 (0, 2) IgE: 2.4 (0.9, 2.7)	SPT: 2 (2,2.5) IgE: 3.4 (2.5, 4.3)	SPT: 0 (0, 1) IgE: 1.8 (0.7, 2.5)	SPT: 0.5 (0, 1) IgE: 2.4 (2.1, 2.8)
	Eats without reaction	*n*	6	5	5	4
		SPT Median (25th–75th IQR) IgE kUA/L median (25th‐75th IQR)	SPT: 0 (0,2) IgE:0.5 (0.3,1)	SPT: 0 (0,3) IgE: 1.8 (1.6,2)	SPT: 0.5 (0,2) IgE (0.5, 4.77)	SPT: 2.5 (1,4)

There were children who did not have an OFC as they either had clinical reactions suggestive that their allergy was ongoing or had SPT/sIgE results above the study cutoffs (see Table 3). Interestingly, some patients had positive legume OFCs when challenged at the time of diagnosis or during their follow‐up visits, despite having low SPT or sIgE results at the time of OFC. Among those challenged, positive OFC outcomes occurred in 38.5% (5/13) for lentil, 33.3% (2/6) for chickpea, 28.6% (4/14) for pea, and 12.5% (1/8) for bean (Table 3).

Further analyses were performed to determine patient variables predictive of the persistence or resolution of NPL allergies (Table 4). The only significant association was between children with a history of anaphylaxis to peas and persistent pea allergy, of which none of these children outgrew their allergy. For lentil allergy, resolution was less frequent in those with multiple NPL allergies (2.5%) than those with single or dual allergies (11.9% and 15%). A similar pattern was seen for chickpea (5.1% vs. 18.7% and 17.9%), whereas pea allergy showed higher resolution in multiple NPL allergy (25.6%) compared to single or dual allergies (16.7% and 4.8%). These associations were not statistically significant. Interestingly, in bean allergy, 84.6% of children with single NPL allergy outgrew it, while none with dual or multiple NPL allergies did, which was statistically significant.

**TABLE 4:** Comparison between children who had persistent or outgrown non‐priority legume allergies.

Factors		Lentil			Chickpea			Pea			Bean		
		Persistent	Outgrown	χ^2^ (CI)	Persistent	Outgrown	χ^2^ (CI)	Persistent	Outgrown	χ^2^ (CI)	Persistent	Outgrown	χ^2^ (CI)
		*N* = 125 (%)	*N* = 14 (%)	*p* Value	*N* = 74 (%)	*N* = 9 (%)	*p* Value	*N* = 69 (%)	*N* = 15 (%)	*p* Value	*N* = 35 (%)	*N* = 11 (%)	*p* Value
Male		78 (88.6)	10 (11.4)	0.43 (0.45–5.07) .5	44 (91.7)	4 (8.3)	0.82 (0.13–2.12) .36	44 (78.6)	12 (21.4)	1.46 (0.59–8.83) .22	21 (75)	7 (25)	0.05 (0.29–4.74) .83
Atopic dermatitis		119 (90.8)	12 (9.2)	0.15 (0.07–5.88) .7	68 (88.3)	9 (11.7)	0.66 (0.88–0.99) .42	63 (81.8)	18 (18.2)	0.07 (0.15–11.9) .79	34 (75.6)	11 (24.4)	0.32 (0.92–1.03) .57
Allergic rhinitis		96 (88.9)	12 (11.1)	0.58 (0.38–8.57) .44	14 (87.5)	2 (12.5)	0.047 (0.16–4.1) .82	58 (82.9)	12 (17.1)	0.15 (0.18–3.13) .7	25 (73.5)	9 (26.5)	0.47 (0.33–9.8) .49
Asthma		16 (88.9)	2 (11.1)	0.01 (0.31–3.57) .88	23 (85.2)	4 (14.8)	0.71 (0.14–2.3) .44	52 (83.9)	10 (16.1)	0.48 (0.19–2.18) .49	26 (76.5)	8 (23.5)	0.01 (0.2–4.25) .92
Other Food allergies		124 (89.9)	14 (10.1)	0.11 (0.98–1.01) .74	72 (88.9)	9 (11.1)	0.13 (0.96–1.01) .72	67 (81.7)	15 (18.3)	0.45 (0.93–1.01) .51	33 (75)	11 (25)	3.52 (0.61–0.9) .42
History of Anaphylaxis		13 (92.9)	1 (7.1)	0.15 (0.08–5.49) .7	14 (100)	0 (0)	2.08 (0.72–0.9) .15	15 (100)	0 (0)	**3.97 (0.69–0.89)** .**04**	9 (100)	0 (0)	0.66 (0.87–102) .06
Single NPL allergy		52 (88.1)	7 (11.9)	3.8 .15	13 (81.3)	3 (18.7)	3.3 .19	20 (95.2)	1 (4.8)	4.1 .13	2 (15.4)	11 (84.6)	36.7 .001
Dual NPL allergies		34 (85)	6 (15)		23 (82.1)	5 (17.9)		20 (83.3)	4 (16.7)		11 (100)	0 (0)	
Multiple NPL allergies		39 (97.5)	1 (2.5)		37 (94.9)	2 (5.1)		29 (74.4)	10 (25.6)		22 (100)	0 (0)	
SPT Median (25th–75th IQR)	Initial diagnosis	8 (3, 12)	0 (0,11)	.17	5 (2, 6)	1 (0, 4)	.**02**	5 (3, 8)	4 (3, 5)	.22	7 (4–8)	3 (1–8)	.26
	At age of 5	8 (4, 13)	0 (0, 0)	.**038**	4 (3, 8)	2 (0, 3)	.2	5 (4, 8)	1 (0, 2)	.**006**	5 (5, 9)	1 (0, 1)	.**006**
	At age of 10	7 (5, 10)	2 (0, 3)	**<.001**	7 (4, 9)	1 (0.5)	.08	4 (3, 7)	0 (0, 0)	**<.001**	7 (3, 10)	0 (0, 1)	.**02**
IgE Median (25th–75th IQR)	Initial diagnosis	9 (3, 35)	3 (1, 5)	.**002**	17 (5, 34)	3 (0, 4)	.**05**	11 (2, 23)	2 (2, 4)	.13	NA	NA	NA
	At age of 5	9 (4, 24)	0 (0, 1)	.**009**	18 (8, 32)	2 (2, 2)	.**02**	13 (4, 25)	2 (2, 3)	.**01**	8 (4, 22)	2 (2, 3)	.35
	At age of 10	20 (11, 30)	2 (1, 3)	**<.001**	20 (8, 30)	5 (1, 21)	.36	13 (6, 34)	1 (1, 1)	.**002**	NA	NA	NA

Bold values indicate statistical significance (*p* < .05).

SPT wheal sizes and sIgE levels at initial diagnosis, ages 5 and 10 were compared in children with persistent and those who outgrew NPL allergies. SPT and sIgE were lower in the outgrown group compared to the persistent group among all NPLs. For lentil allergy, SPT size was significantly lower in the outgrown group at ages 5 and 10, while sIgE levels were significantly lower at all time points. For chickpea allergy, SPT size at initial diagnosis was significantly lower in the children who outgrew their allergy, while sIgE levels at initial diagnosis and age 5 were significantly lower with subsequent allergy resolution. For pea allergy, SPT size and sIgE levels were significantly lower in the children who outgrew allergy at age 5 and 10 years. For bean allergy, bean SPT size was significantly lower with allergy resolution at ages 5 and 10. These suggest that lower SPT or sIgE values may be predictive of NPL allergy resolution (Table 4).

## 4 DISCUSSION

Plant‐based foods such as lentils, chickpeas, peas, and beans have become more popular in diets globally, but with this we have seen an increase in FA to these foods as well. The data for these emerging FA is limited, and therefore warrants further investigation to understand better the epidemiology of legume allergies.

The prevalence rate of NPL in this cohort was 6.3% in a UK pediatric allergy clinic among the food allergic population. This is a notably small proportion compared to 25% of children with FA who have peanut allergy.^15^ There were more male children who had legume allergies in our population (62.6%), which aligns with broader FA trends, where males have been reported to have a higher prevalence of FA in childhood.^1^, ^16^ A Canadian study highlighted a similar trend with a disproportionate representation of males, accounting for 64.6% of cases of NPL allergy.^17^

Our study found a high prevalence of atopic disorders among patients with NPLs. Nearly all of the children had other food allergies and the majority of them have atopic disorders, as would be expected in a tertiary care setting.^18^, ^19^ This raises the possibility of selection bias, where patients with a single legume allergy are not considered severe enough for a referral to a specialist centre and consequently, the prevalence of these allergies could be under‐recognized in our study. Nevertheless, previous studies in both pediatric and adult populations have shown that single NPL allergy is uncommon, supporting that affected individuals present with multiple NPL allergies.^9^, ^20^ A Spanish study showed that patients with legume allergies are highly atopic, which could be a clinical characteristic of patients allergic to legumes.^21^ In our cohort, lentil allergy was the most common NPL allergy, followed by peas, chickpeas, and beans. These figures are close to other studies that show that the prevalence of lentil allergy is 3.1%–5.9%, chickpea allergy is 2.3%–3.8%, pea allergy is 1.5%–2.3%, and bean allergy is 1.8%.^22^

This study indicated that 20.7% of the children had experienced anaphylaxis to NPL at some point, and 10.8% self‐reported a reaction from inhalant exposure of a NPL. A study from EAR across all age groups reported an estimate of 1.6% of food‐induced anaphylaxis cases attributed mainly to NPLs. Another study from EAR analyzed 1970 cases of anaphylaxis in children and showed that 5% were related to NPLs and lupine.^13^, ^23^ Although information about food reactions through inhalation is limited, there are reports of allergic reactions after exposure to airborne particles from NPLs with manifestations of urticaria, angioedema, allergic rhinitis, and asthma exacerbations.^24^

This study revealed a high level of cross‐reactivity among the NPLs, where children with one NPL allergy were likely to have others. These findings underscore a strong immunological linkage across these legumes, consistent with other studies in Spain and Turkey that reported that a significant majority of children with legume allergies reacted to multiple NPLs, particularly with lentils, peas, and chickpeas.^12^ Cross reactivity among NPL allergies is probably attributed to vicillins, profilins, and non‐specific lipid transfer proteins (ns‐LTPs).^21^ Vicillins are heat‐stable storage proteins, making these legumes allergenic even after cooking and contributing to cross‐reactivity with other legumes like peanuts. Profilins are heat‐sensitive proteins in plants and pollens that often trigger OAS and are observed in 8.9% of the children in this study. ns‐LTPs are a family of stable proteins that are resistant to heat and digestion, making them major triggers of severe allergic reactions, including anaphylaxis and exercise‐induced anaphylaxis.^25^

Similarly to our results, a Turkish study reported a median age of diagnosis of approximately 19 months, with a predominance of male patients. More than 90% of the children had coexisting food allergies, and nearly 90% had atopic comorbidities. Additionally, around 20% had a history of anaphylaxis.^26^ Lentil was the most common allergen (66%), followed by peanut (61%), chickpea (28%), pea (24%), and bean (8%). While single lentil allergy was 16% of cases, no cases of isolated allergy to chickpea, pea, or bean were observed. Notably, 60% of the patients had multi‐legume allergies, suggesting a high degree of cross‐reactivity among NPL.^26^

This study tracked the resolution of NPL allergies in children and showed increasing tolerance rates as they age. At age 15, the resolution rates for lentils, chickpeas, peas, and beans were 21%, 19.3%, 23.5%, and 32.9%, respectively. A Turkish study showed that 50% of patients had outgrown their lentil allergy before their teenage years, though it had a small sample size of only 30 participants.^27^ Another study, which included participants with peanut, soy, and non‐priority legume allergies, reported a 44.4% likelihood of achieving tolerance among patients allergic to one legume.^28^ In comparison to priority legumes, a Canadian study showed that peanut resolution rates were 10% at age 4, 26% at age 10, and 27% at age 12.^29^ An Australian study showed higher resolution rates of peanut allergy of 21.4% at age 5 and 34.2% at age 7.^30^ A USA study reported the resolution of soy allergy in 25% by age 4 years and 69% by age 10 years.^31^ The resolution rates of NPL allergies observed in our study are generally lower, particularly for lentils and chickpeas.

Our study findings indicate that children with single NPL allergy have a better chance of NPL allergy resolution compared to those with dual and multiple NPL allergies. This association reached statistical significance for bean allergy but not for other NPL allergies. In comparison, a study showed that children with multiple sensitizations were more likely to experience persistent peanut and egg allergies.^32^ In addition, our study showed that the risk of anaphylaxis was highest among children with multiple NPL allergies, followed by those with dual and then single NPL allergies. This is consistent with a study that exhibited children with multiple food allergies having more than threefold higher odds of experiencing severe allergic reactions compared to those with a single food allergy.^33^

We also found an association between lower SPT and sIgE levels in predicting resolution or persistence of NPL allergies. For all the NPL allergens, children with lower SPT and sIgE levels at ages 5 and 10 were more likely to outgrow these allergies. There is some evidence that suggests that lower sIgE at diagnosis and decreasing sIgE levels or SPT wheal size over time predict FA resolution.^34^ A study revealed that peanut biomarkers measured at diagnosis did not help predict peanut allergy resolution; a decrease in Ara h 2 sIgE over time was associated with an increased likelihood of outgrowing peanut allergy.^35^ A study found that decreasing levels of sIgE were significantly associated with an increased likelihood of developing clinical tolerance to both egg and milk in children.^36^

This study highlights a discrepancy in the outcomes of legume allergy challenges, where many patients exhibited allergic reactions despite having low SPT or sIgE results. While these tests, if low, are often used to determine suitability for OFCs, these tests may only partially predict reactions. Therefore, it is essential to consider a broader range of factors within the patient's clinical context, such as age, time since last reaction, and importance of the food in the child's diet before deciding to conduct an OFC.^34^

### 4.1 Limitations

The study's limitations include its retrospective design, which is inherently limited by the reliability of data originally collected. Thus, the accuracy and completeness of the medical records dictate the quality of the data available for analysis. There is also a likely degree of selection bias, as the population were referred to a specialist centre where there is a greater likelihood for children with multiple food allergies, multiple atopic comorbidities and more severe reactions to be seen. This limits the generalizability of our results to a wider UK population. Additionally, not all children had their NPL original diagnosis confirmed by OFC, meaning some cases were based on parental‐reported allergic reactions rather than objective confirmation. Therefore, the interpretation of persistent allergy must also be considered as given the high degree of cross‐sensitization among legumes, elevated SPT or sIgE results may not always represent true clinical persistence but rather cross‐reactivity with another legume. This could have led to overestimation of persistent allergy in some cases. Another limitation is the lack of a standardized definition of tolerance, as there was no consistent measure of how often or in what quantity the allergenic food was consumed to determine resolution. An important additional limitation relates to diagnostic testing: standardized commercial SPT extracts have very poor sensitivity for legume allergies, especially lentil. In clinical practice, prick‐to‐prick testing with the actual food allergen is often preferred for diagnosing these allergies. Consequently, if these aren't used, they generally come up with low numbers even when the kids are allergic.^37^ Prospective studies are needed to further our understanding of the nature of NPL allergies.

## 5 CONCLUSION

Non‐priority legumes are an increasing cause of allergy, yet data about their epidemiology is limited. Our study found that 6.3% of children with FA have an allergy to NPLs, with lentil allergy being most prevalent, and approximately 20% of children have experienced anaphylaxis to NPLs. The rate of resolution of legume allergies is around 20%–32.9% by age 15, a pattern not dissimilar to that observed with peanut allergy resolution. Lower SPT and/or sIgE levels were associated with a higher likelihood of allergy resolution.

## AUTHOR CONTRIBUTIONS

**Ali Atwah:** Methodology; data curation; project administration; formal analysis; writing – original draft. **Kate Swan:** Methodology; project administration; writing – review and editing; supervision. **Adam T. Fox:** Conceptualization; methodology; writing – review and editing; supervision. **Ru‐Xin Foong:** Methodology; supervision; writing – review and editing; project administration.

## FUNDING INFORMATION

The authors received no funding for this work.

## CONFLICT OF INTEREST STATEMENT

The authors declare no conflicts of interest.

## USE OF ARTIFICIAL INTELLIGENCE (AI) AND AUTHORSHIP

Artificial intelligence tools were used solely to generate Figure 1 using Napkin.ai. AI tools were not used in the conception, study design, data analysis, interpretation of results, or writing of the manuscript.

## Supporting information


Data S1.

## Data Availability

The data supporting the findings of this study are available from the corresponding author upon reasonable request.
