# Supplementary material for: The prevalence of outgrowing non‐priority legume allergies in children
Source: Pediatr Allergy Immunol. 2025 Dec 22;36(12):e70269. doi: 10.1111/pai.70269 (PMC12720222; doi:10.1111/pai.70269)
Supplement: Supplementary file 1 — Data S1. [file PAI-36-e70269-s001.docx]

**Supplementary Appendix**

**S1: Additional information for the Methodology section**

**Skin Prick Test and Specific IgE tests**

SPT was performed using fresh food for lentil, chickpea, pea, and bean. Histamine and saline were used as positive and negative controls, respectively. sIgE testing was performed using the Thermofisher platform. SPT and sIgE tests were performed for one or more NPLs based on reported symptoms that suggest IgE-mediated allergy. Routine screening for all four NPLs was not conducted for every patient, and if a child consumes a specific NPL without symptoms, further testing for that legume was typically not pursued. Testing methods and strategy remained consistent for all children presented to the paediatric allergy department.

**Missing Data**

Patients who visit the clinic usually have either SPT or sIgE tests. SPT is the standard initial test for most patients; if performed and the result exceeds the diagnostic cutoff, a sIgE test is usually not pursued. However, in cases where patients were taking antihistamines, had SPT results below the diagnostic cutoff, or declined SPT, sIgE testing was used. This approach resulted in numerous missing data points among SPT or sIgE results. To deal with missing data, a sensitivity analysis was used to address missing data in SPT and sIgE results. First, the analysis was conducted on complete cases using listwise deletion. Then, multiple imputation was performed to handle missing data, and the analysis was repeated on the imputed datasets. Results from both methods were compared, and as no significant differences were found, the complete case analysis was retained for the final results.

**Table S1: Proportion of Missing Values and Imputed Data for Skin-Prick Test (SPT) and Specific IgE Results to Lentil, Chickpea, Pea, and Bean at Different Time Points**

| **Categories** | **Missing** | **Total** | **Percentages** | **Imputed** |
| --- | --- | --- | --- | --- |
| **SPT Lentil at time of diagnosis** | 55 | 139 | 39.56 | Yes |
| **IgE lentil at time of diagnosis** | 88 | 139 | 63.3 | Yes |
| **SPT to lentil at 5** | 70 | 139 | 50.35 | Yes |
| **IgE to lentil at 5** | 93 | 139 | 66.9 | Yes |
| **Lentil SPT at Ten** | 90 | 139 | 64.7 | Yes |
| **Lentil IgE at Ten** | 109 | 139 | 78.4 | Yes |
| **Lentil SPT Last visit** | 34 | 139 | 24.46 | Yes |
| **Lentil IgE Last vist** | 76 | 139 | 54.6 | Yes |
| **Spt Chickpea at time of diagnosis** | 35 | 83 | 42.17 | Yes |
| **IgE chickpea at time of diagnosis** | 52 | 83 | 62.65 | Yes |
| **SPT to Chickpea at 5** | 44 | 83 | 53.01 | Yes |
| **IgE to Chickpea at 5** | 51 | 83 | 61.44 | Yes |
| **Chickpea SPT at Ten** | 52 | 83 | 62.65 | Yes |
| **Chickpea IgE at Ten** | 65 | 83 | 78.31 | Yes |
| **chickpea SPT Last visit** | 20 | 83 | 24.09 | Yes |
| **Chickpea IgE Last visit** | 43 | 83 | 51.8 | Yes |
| **SPT pea at time of diagnosis** | 44 | 84 | 52.38 | Yes |
| **IgE Pea at time of diagnosis** | 48 | 84 | 57.14 | Yes |
| **SPT to Pea at 5** | 42 | 84 | 50 | Yes |
| **IgE to Pea at 5** | 52 | 84 | 61.9 | Yes |
| **Pea SPT at Ten** | 50 | 84 | 59.52 | Yes |
| **Pea IgE at Ten** | 55 | 84 | 65.47 | Yes |
| **Pea SPT Last visit** | 21 | 84 | 25 | Yes |
| **Pea IgE Last visit** | 25 | 84 | 29.76 | Yes |
| **SPT bean at time of diagnosis** | 34 | 46 | 73.91 | Yes |
| **IgE Bean at time of diagnosis** | 38 | 46 | 82.6 | No |
| **SPT to Bean at 5** | 27 | 46 | 58.6 | Yes |
| **IgE to Bean at 5** | 39 | 46 | 84.78 | No |
| **Bean SPT at Ten** | 37 | 46 | 80.43 | Yes |
| **Bean IgE at Ten** | 41 | 46 | 89.13 | No |
| **Bean SPT Last visit** | 24 | 46 | 52.17 | Yes |
| **Bean IgE Last visit** | 31 | 46 | 67.39 | Yes |
